# Laparoscopic surgery for impacted dentures in the descending colon: A case report

**DOI:** 10.1016/j.ijscr.2025.111141

**Published:** 2025-03-13

**Authors:** Trung Nguyen Vo, Tung Viet Le, Vinh Quoc Nguyen, Thanh Tan Nguyen

**Affiliations:** a Department of Clinical Pathology, University of Medicine and Pharmacy at Ho Chi Minh City, Viet Nam; b Training and Scientific Research Department, University Medical Center Ho Chi Minh City, Ho Chi Minh City, Viet Nam; c Department of General Surgery, University Medical Center Ho Chi Minh City, Ho Chi Minh City, Viet Nam; d Faculty of Public Health, University of Medicine and Pharmacy at Ho Chi Minh City, Ho Chi Minh City, Viet Nam; e Faculty of Medicine, University of Medicine and Pharmacy at Ho Chi Minh City, Ho Chi Minh City, Viet Nam

**Keywords:** Ingestion of foreign bodies, Ingestion of dentures

## Abstract

**Introduction and importance:**

Denture ingestion, commonly seen in older adults, can also occur in younger individuals. Most dentures require intervention as they cannot be excreted naturally. We report a case of a young male undergoing laparoscopic surgery to remove dentures impacted in the descending colon two months post-ingestion.

**Case presentation:**

A 30-year-old male presented with intermittent left lower quadrant abdominal pain for three days. He had accidentally swallowed his dentures two months earlier. Abdominal X-ray showed a radiopaque foreign body in the descending colon. Endoscopic retrieval attempts failed, necessitating urgent surgery. The dentures were removed successfully through laparoscopic surgery, and the perforation was closed using continuous horizontal PDS 4.0 sutures. The postoperative course was uneventful and the patient was discharged on postoperative day four.

**Clinical discussion:**

Denture ingestion poses a significant risk due to the nature of the object, often requiring removal as it cannot pass through the gastrointestinal tract. Removable dentures are a known risk factor for such accidents. Diagnosis typically involves abdominal X-rays or CT scans. Endoscopic retrieval is often attempted first but carries a risk of perforation, which may require emergency surgical intervention.

**Conclusion:**

Surgical approaches depend on the location and extent of perforation and the patient's abdominal condition. Early laparoscopic intervention should be considered in cases where surgery is indicated, as it offers a minimally invasive and effective solution.

## Highlights

• The use of removable dentures is a risk factor for involuntary ingestion of dentures.
• Dentures can remain in the digestive tract for a long time after ingestion.
• Diagnosis relies on abdominal X-rays or computed tomography scans depending on the characteristics of the dentures.
• Ingestion of dentures often requires intervention for removal.
• Laparoscopic surgery should be chosen in situations where surgical intervention is warranted.

## 1 Introduction and importance

Accidental or intentional ingestion of foreign bodies is a relatively common situation in everyday life. Most foreign bodies can pass through the digestive tract and be excreted through the rectum smoothly approximately 4–6 days after swallowing, and about 1 % of foreign bodies can cause complications such as intestinal perforation [[1], [2], [3]]. In the United States, an estimated 1500 people die each year from ingesting foreign bodies [4]. Factors that contribute to foreign bodies getting stuck in the digestive tract include anatomical features of the digestive tract such as narrow passages, angles, diverticula, or previous surgeries, as well as the characteristics of the foreign bodies.

Ingested foreign bodies often vary in nature, including blunt objects, sharp objects, medication capsules, and medical materials (dentures, inactive endoscopic capsules, etc.). Among these types of foreign bodies, dentures, especially removable dentures, which can be detached, have been reported as the most commonly accidentally ingested foreign bodies [5,6]. Ingestion of dentures is more common in older individuals but can also occur in younger people [7]. We report a case of a young individual accidentally ingesting dentures and undergoing laparoscopic surgery to remove the dentures impacted in the descending colon 2 months after ingestion. The cases reported in the literature are reviewed and the treatment of ingestion of dentures is also discussed.

## 2 Case presentation

**Fig. 1:**
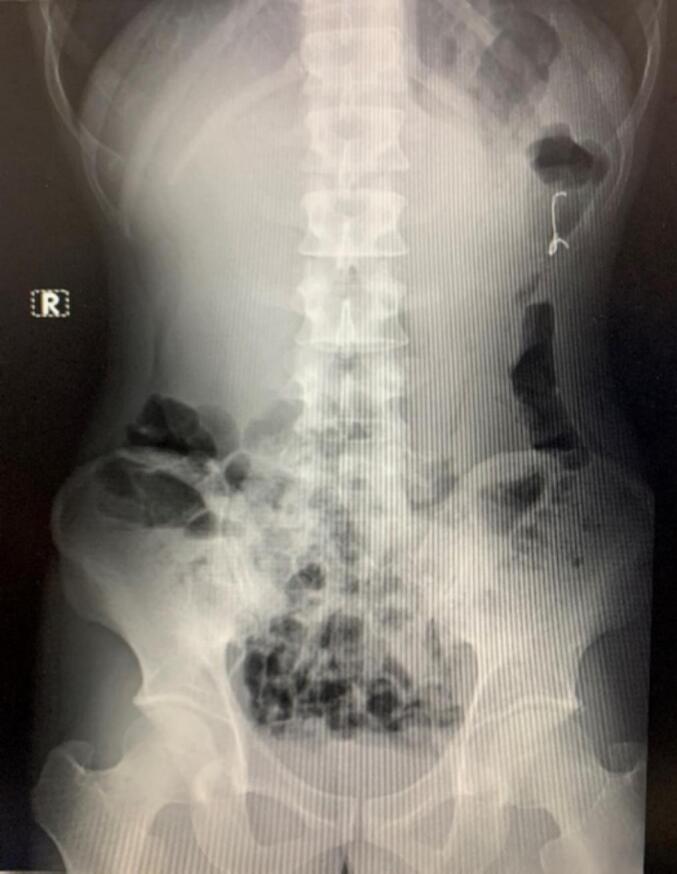
The abdominal X-ray upon admission showed a foreign body impacted in the descending colon.

**Fig. 2:**
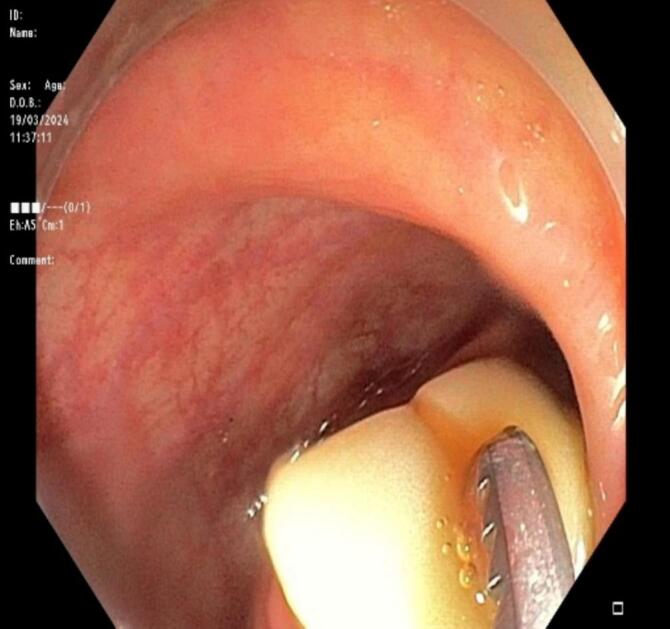
Colonoscopy identified the foreign body as dentures impacted in the descending colon.

**Fig. 3:**
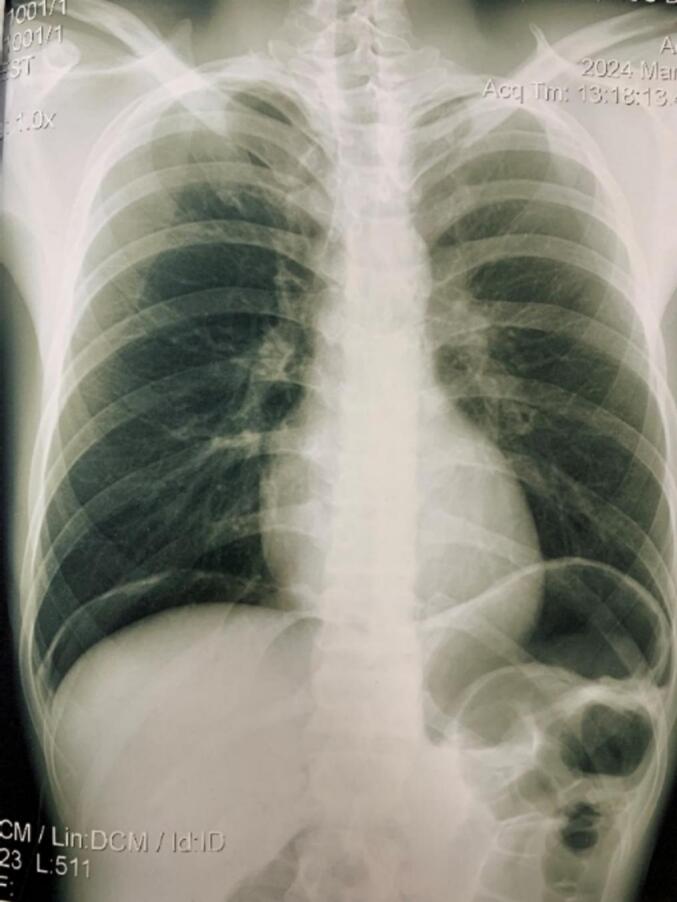
The chest X-ray after the colonoscopy showed a free air under the right diaphragm.

A 30-year-old male patient presented with intermittent abdominal pain in the left lower quadrant for about 3 days. Two months prior to admission, the patient accidentally swallowed his dentures. After ingesting the dentures, the patient did not seek any treatment. Two weeks later, he occasionally experienced crampy abdominal pain with changing locations. He went to the local hospital, where an X-ray was performed and identified dentures in the small intestine. He was advised to wait for the dentures to pass naturally with bowel movements. The patient's past medical history was unremarkable. On the day of admission, the patient experienced significant abdominal pain on the left side and sought medical attention. On abdominal examination, the abdomen was soft, non-tender, and non-distended. An abdominal X-ray revealed a radiopaque foreign body in the descending colon and no free air in the peritoneal cavity (Fig. 1). The patient was diagnosed with dentures impacted in the descending colon and was scheduled for a colonoscopy to remove the dentures. During the colonoscopy, we observed that the dentures were impacted in the descending colon and proceeded to grasp the dentures (Fig. 2). However, retrieving the dentures via endoscopy was very challenging, and the colonic mucosa was significantly scratched, so we decided to stop the procedure and plan for surgery. A preoperative chest X-ray showed subdiaphragmatic free air on the right side (Fig. 3). Based on this result, we suspected a situation where the dentures had perforated the colon and decided to perform emergency surgery. Preoperative blood tests showed a white blood cell count of 8520/mm^3^, with neutrophils accounting for 63.6 %. Other biochemical tests were within normal limits.

**Fig. 4:**
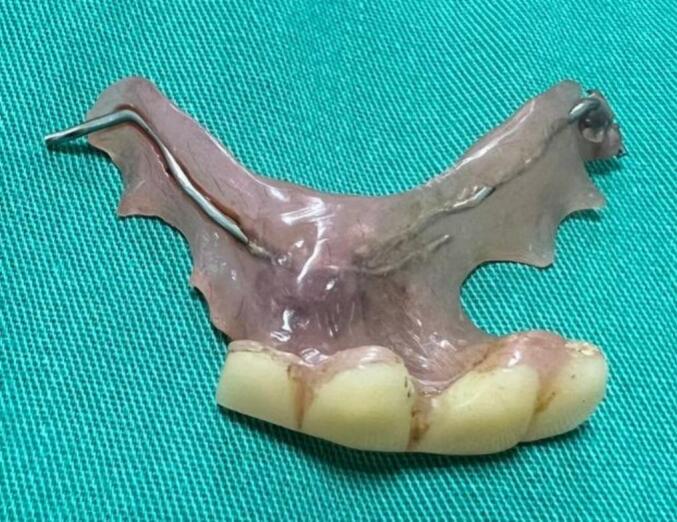
The dentures after removal.

Laparoscopic surgery was performed after endoscopic retrieval attempts. During the surgery, we observed turbid fluid in the descending colon. There was a 1 cm perforation in the descending colon near the splenic flexure, exactly where the dentures were located (Video 1, Fig. 4). We enlarged the perforation to remove the dentures and closed the perforation with continuous horizontal PDS 4.0 sutures. Finally, we thoroughly irrigated the abdominal cavity, placed a drain near the descending colon, placed the dentures in a bag, and removed it through a small incision. Postoperatively, the patient underwent abdominal examinations twice a day, oral feeds resumed on the second postoperative day, and laboratory tests and ultrasound were done on the 3rd postoperative day. The patient recovered uneventfully and discharged on the 4th postoperative day.

## 3 Method

This case report adheres to the SCARE criteria [3,8,9].

## 4 Discussion

### 4.1 Physiology and evolution of ingestion of dentures

Ingestion of foreign bodies can occur accidentally or intentionally. Accidental ingestion is more common in children, adolescents, the elderly, alcohol or drug addicts, visually impaired individuals, those with psychiatric disorders, and people who frequently put small objects in their mouths as part of their occupation (such as carpenters, tailors, and upholsterers), as well as those who have a habit of eating very quickly [1,10]. Intentional swallowing is often seen in cases of prisoners in correctional facilities, suicide attempts, psychiatric patients, and drug traffickers [11]. A risk factor for ingestion of foreign bodies is the use of dentures, especially removable ones. Rodrıguez-Hermosa's study showed that 72.7 % of cases of foreign body ingestion involved the use of dentures or dental appliances [12].

After ingestion, foreign bodies are often impacted at the upper/lower esophageal sphincter, pylorus, duodenum, ileocecal valve, caecum, sigmoid, and anus [13]. The locations where foreign bodies often cause perforation are the ileocecal and the rectosigmoid regions due to the narrowing of the bowel lumen and angulation of the digestive tract in these areas [12,14]. Dentures are often made of non-radiopaque plastic, some types may have metal clasps, and this is the main reason for dentures getting trapped or causing gastrointestinal perforation [15]. Theoretically, most cases of dentures passing through the ileocecal valve will easily pass through the colon. Moreover, the density of feces can sometimes even form a protective barrier against injury to the colon. Webb's study of 192 cases of foreign body ingestion showed that most foreign bodies are spontaneously expelled, about 10–20 % require non-surgical intervention, and less than 1 % require surgical intervention [3]. Although our case had metal clasps, they still passed through the ileocecal valve and were impacted in the descending colon, causing pain. However, since we performed endoscopic intervention to remove the dentures, it is uncertain whether it could continue to move down to the rectum on its own.

The time from ingesting the foreign bodies to the appearance of symptoms is often prolonged (more than 2 weeks in 50 % of cases and many months or even years in some cases), so patients often do not associate their symptoms with the ingestion of foreign bodies [14,16]. Flanagan reported a case of denture impaction in the sigmoid that was discovered 3 years after ingestion [17]. In our case, the patient presented for examination 2 months after ingestion of dentures and informed the doctor about the incident, so the initial diagnosis was not difficult.

### 4.2 Diagnosis

The clinical symptoms of foreign body ingestion vary from abdominal pain to peritonitis or bowel obstruction depending on the extent of injury caused by the foreign bodies. In our case, as the dentures had not perforated the colon, the patient only experienced abdominal pain and showed no signs of peritonitis. Imaging modalities are very useful in locating the position of the foreign bodies. The choice of diagnostic imaging modality depends on the characteristics of the foreign bodies, the time of ingestion, and the patient's clinical symptoms. Dentures are typically made of non-radiopaque material, but some types may contain metal hooks that can be detected on conventional abdominal X-rays. If the patient presents to the hospital immediately after ingestion of foreign bodies, esophagogastroduodenoscopy should be performed initially. Some studies suggest that abdominal computerized tomography (CT) is the best imaging modality because it provides detailed information about the location of the foreign bodies, the presence of free air, fat stranding around the foreign bodies, or signs of bowel obstruction [14,16]. Free air may not be seen on conventional abdominal X-rays because the perforation site is sealed by foreign bodies, fibrin, mesentery, or adjacent organs, preventing the leakage of gas or intestinal fluid into the peritoneal cavity [14,16]. In our situation, the patient presented to the hospital 2 months after ingestion of dentures and did not exhibit clinical symptoms suggestive of infection, therefore we initially performed an abdominal X-ray instead of CT scanning. Due to the presence of metal hooks in the dentures, the initial X-ray confirmed their location in the descending colon; and since there was no evidence of free air in the peritoneal cavity, we decided not to perform CT scanning and proceeded with a colonoscopy to retrieve the dentures.

Diagnosis of foreign body ingestion is usually not difficult if the patient informs the physician about the incident. In cases of acute abdominal pain where the patient does not recall ingesting a foreign body, the diagnosis of an impacted foreign body is often made after obtaining CT scan results. However, if the foreign bodies are not radiopaque, diagnosis can be challenging, and in such situations, a definitive diagnosis is often made during surgery [12,18]. We believe that questioning the patient about the characteristics of the foreign bodies plays an important role in selecting the appropriate imaging diagnostic modality and analyzing imaging results.

### 4.3 Treatment

Currently, there is still no consensus on the treatment protocol for patients who have swallowed foreign bodies because the majority of cases allow the foreign bodies to pass through the digestive tract and be excreted through the rectum spontaneously [19].

Treatment methods for foreign body ingestion vary from observation, medication use, and endoscopic intervention, to surgery. The choice of method depends on the patient's characteristics, the nature and location of the foreign bodies, complications, the facilities of the medical institution, and ultimately the patient's preferences. Foreign bodies can cause complications such as gastrointestinal bleeding, obstruction, perforation, peritonitis, intraabdominal abscess, and even death. The leading cause of death is often severe infection leading to multiple organ failure [1]. Therefore, once complications arise, the treatment method is often surgical intervention [12,20]. Current recommendations for the initial management of foreign body ingestion are conservative treatment, close observation, and continuous imaging monitoring for about 3 days. In cases where conservative treatment fails, efforts should be made to retrieve the foreign bodies via endoscopy or early surgical intervention to avoid the risk of perforation and subsequent complications [21,22]. Goh's study showed that complication and mortality rates in cases of perforation were 24.2 % and 6.5 %, respectively [23]. Gachabayov reported two cases of denture ingestion, in which one patient passed it naturally, while the other required surgical removal [24]. Ganesh studied 34 cases of foreign body ingestion and also suggested that gastrointestinal foreign bodies should be removed as soon as possible to minimize the risk of serious complications [25]. In our case, we decided to intervene and retrieve the dentures based on several factors: First, the patient had swallowed the dentures for 2 months; second, the dentures had metal hooks; third, the narrow location in the descending colon; and finally, the patient had symptoms of pain. All these factors indicated that the dentures were likely to become trapped and would not naturally pass out. For this reason, we decided to perform a colonoscopy to retrieve the dentures. We hoped that by inflating the colon during colonoscopy, we could expand the lumen of the colon and successfully retrieve the dentures. However, due to the characteristics of the dentures, we were unable to retrieve them successfully and accidentally perforated the colon, necessitating emergency surgical intervention.

Some authors have proposed laparoscopy in the diagnosis and treatment of foreign body ingestion [26,27]. In Gachabayov's report, the foreign body was removed through open surgery, while in Ganesh's study, cases of foreign body ingestion were managed using both laparoscopic and open surgery [24,25]. After the foreign bodies have been extracted, different surgical techniques can be performed, such as primary suture, segmental resection with anastomosis, and colostomy [1,28]. The decision to perform a colostomy depends on various factors, such as the characteristics of the injury and the degree of abdominal infection. In Rodriguez-Hermosa's study, 39.4 % required a colostomy [12]. Flanagan reported a case of sigmoidectomy due to ingestion of dentures [17]. In our case, the dentures were successfully retrieved through laparoscopic surgery and our results showed that duration of impaction of dentures did not influence the choice of laparoscopic surgery. Due to timely emergency surgery, the abdominal cavity was clean and the colon had been prepared, we only retrieved the dentures and closed the colonic opening without segmental resection or colostomy.

## 5 Conclusions

Ingestion of dentures is more common in older adults but can also occur in younger individuals. The use of removable dentures can be considered a common risk factor for this accident. Due to their characteristics, dentures are often difficult to pass naturally through the anus and usually require intervention for removal. Diagnosis frequently relies on regular abdominal X-rays and computerized tomography scans, and the choice of method depends on the clinical context. Endoscopic retrieval of the dentures can be performed initially; however, patients should be counseled beforehand about the risk of perforation during the procedure and the need for emergency surgical intervention afterward, especially in cases where the foreign body has persisted for a long time. The surgical technique used depends on the perforation's location, the injury's extent, and the abdominal condition. Laparoscopic surgery is feasible and should be considered initially in situations requiring surgical intervention.

The following is the supplementary data related to this article.

Supplementary Video 1 Supplementary Video 1

## Author contribution

Trung Nguyen Vo, Vinh Quoc Nguyen, and Thanh Tan Nguyen were all equally contributing to the pre-, intra- and postoperative treatment of the patient. The first draft of the manuscript was written by Trung Nguyen Vo and Tung Viet Le commented on previous versions of the manuscript. All authors read and approved the final manuscript.

## Patient consent

Written informed consent was obtained from the patient for publication of this case report and accompanying images. A copy of the written consent is available for review by the Editor-in-Chief of this journal on request.

## Ethical approval

This report was carried out in accordance with the Code of Ethics of the World Medical Association (Declaration of Helsinki) for Experiments in Humans.

This case report got ethical approval from our institution. The patient was given consent form before the surgery.

## Guarantor

Trung Nguyen Vo MD, PhD.

## Research registration number

None.

## Funding

None.

## Conflict of interest statement

The authors declare that they have no known competing financial interests or personal relationships that could have appeared to influence the work reported in this paper.
